# Microfabrication of Net Shape Zirconia/Alumina Nanocomposite Micro Parts

**DOI:** 10.3390/nano8080593

**Published:** 2018-08-05

**Authors:** Hany Hassanin, Mahmoud Ahmed El-Sayed, Amr ElShaer, Khamis Essa, Kyle Jiang

**Affiliations:** 1 School of Mechanical and Automotive Engineering, Kingston University, Kingston upon Thames KT1 2EE, UK; 2 Department of Mechanical Design, Faculty of Engineering, Mataria, Helwan University, P.O. Box 11718, Helmeiat-Elzaton, Cairo, Egypt; 3 Department of Industrial and Management Engineering, Arab Academy for Science and Technology and Maritime Transport, Abu Qir, Alexandria 21599, Egypt; m_elsayed@aast.edu; 4 Drug Discovery, Delivery and Patient Care (DDDPC), School of Life Sciences, Pharmacy and Chemistry, Kingston University London, Kingston upon Thames KT1 2EE, UK; A.Elshaer@kingston.ac.uk; 5 School of Engineering, University of Birmingham, Birmingham B15 2TT, UK; K.E.A.Essa@bham.ac.uk (K.E.); k.jiang@bham.ac.uk (K.J.)

**Keywords:** soft lithography, dispersion, alumina, zirconia, nanocomposite, micro fabrication

## Abstract

Recently, there are growing demands in manufacturing of net shape micro parts for wide range of applications due to the increasing interest in miniaturization. In this paper, the fabrication of tetragonal phase zirconia/alumina (YSZ/Al_2_O_3_) nanocomposite micro-parts with high quality is presented. The fabrication process is based on soft lithography and colloidal powder dispersion. Experimental results showed that by optimizing the soft lithography and the dispersion process, it was possible to produce high-resolution micro-parts with well dispersed alumina. The X-ray diffraction results had confirmed the important role of the alumina particles in eliminating the emergence of monoclinic phase while the microstructures reveal a pure tetragonal phase. In addition, the sintered YSZ/Al_2_O_3_ micro parts achieved micro hardness with 20% superior to the pure YSZ sintered micro-parts with the addition of 5% alumina.

## 1. Introduction

Micro-electro-mechanical systems (MEMS) can be manufactured using a wide range of technologies such as by micro machining of silicon-based materials or by photolithography of a UV sensitive resin. However, ceramic materials are also useful alternative, especially in applications such as biomedical micro devices and implants, gas sensors, cutting tools, and components for harsh environments and at high temperatures application [1,2,3]. State-of-the-art ceramic micro devices and platforms include few attempts to fabricate ceramic nanocomposite MEMS using the latest advances in nanomaterials, which have made it possible to control the ceramic materials at nano-scale [4,5,6]. Nanomaterials are materials with sub-micron, which have an increasingly wide range of interesting unique mechanical, electronic and optical properties. On the other hand, the term “nanocomposite” is widely employed to describe multiphase materials, where at least one material is a nanomaterial.

Zaman and others [7] used electrophoretic deposition to fill micro moulds with Boehmite/multi-wall carbon nanotube (CNT) nanocomposite in order to fabricate complex shape micro-gears. The micro moulds were fabricated using rapid proto typing and were filled with stable colloidal suspensions of Boehmite/multi-wall CNT nanocomposite. A homogeneous solution of CNT-boehmite was obtained using hydrothermal treatment, which helped in functionalization of CNTs surface. The colloidal method was recently used to produce alumina toughened zirconia (10 mol.% CeO_2_ was used as stabilizer) nanocomposites with different Al_2_O_3_ additions. The strength was improved from 900 to 930–1100 MPa and the toughness was doubled from 6 to 12.5–13 MPa·m^1/2^ with the addition of 35% and 50% alumina, respectively. Nevertheless, the micro gears produced using this technology were not free standing and the geometry quality was far from being defined as near net shape components. In addition, the tetragonal phase could not be preserved in the surface without grinding the samples after sintering. This stood as a significant limitation especially if the nanocomposites were intended to be used as structural materials [8]. Chan et al. [9] had run some trials to optimize the sol-gel casting technique for the production of silica titania composite micro gears. However, the impermeability of the mould bottom surfaces, that resisted the uniform contraction of the micro gear, was associated with porosity in the bottom surfaces.

Yttria stabilized zirconia (YSZ) was reported to have superior electrochemical, biomedical and mechanical properties. YSZ was reported to have a high affinity to undergo structural changes from the tetragonal phase to the monoclinic one (*t* ⇨ *m*). The transformation provides a toughening mechanism which indirectly leads to strengthening [10,11]. As bulk components, zirconia–alumina nanocomposites have been developed to produce composites with superior properties. One of the early researches to produce Al_2_O_3_-ZrO_2_ system was carried out by Nawa et al. who produced nanocomposites by using ZrO_2_ as the matrix and Al_2_O_3_ as the disperse phase [12]. The addition of 30 vol.% Al_2_O_3_ to Ce stabilized ZrO_2_ allowed the composite to achieve a fracture toughness of 9.5 MPa·m^1/2^. In another study, Benavente and colleagues reported the production of Al_2_O_3_-ZrO_2_ nanopowder compacts using microwave-assisted sintering. The authors concluded that the sample with 10 vol.% ZrO_2_ had the highest Vickers hardness (20 GPa) with Young’s modulus values of 367 GPa [13]. Although there was a plenty of research on the fabrication of YSZ micro components for wide range of micro system applications, the material properties need to be further improved with the use nanocomposite route. To the best of the authors’ knowledge, there is no reported work on the fabrication and the characterization of zirconia-alumina nanocomposite micro parts to further improve its microstructural and hardness property of YSZ. Therefore, the objective of the work is to explore the feasibility of using a modified soft lithography and colloidal powder processing to develop near net shape YSZ/alumina nanocomposite micro parts with improved properties and to define the role of the alumina addition in the properties of the sintered nanocomposite micro parts.

Soft lithography represents a cheap and single step technique for ceramic micro fabrication [14,15]. In this process, a model is designed and printed on a transparent polymer with a commercial image setter. Soft moulds from polydimethylsiloxane (PDMS) are often used for patterning the desired geometry. This is followed by micro moulding (stamping), replication of ceramic green micro-parts and subsequent sintering. Through this technique, complete filling of the micro patterns is guaranteed, which represents a distinguished merit for fabricating ceramic micro-parts through the suspension route. Moreover, the process is relatively rapid; the whole procedure from design to manufacture completes within 24 h [16,17,18,19,20]. In the present paper, colloidal powder processing in combination with soft lithography is introduced as a robust forming technique for the fabrication of stabilized zirconia/alumina nanocomposites micro-parts. The research is intended to obtain net shaped micro-parts of homogenously dispersed alumina nanoparticles into stabilized zirconia matrix. Suspension dispersion of both stabilized zirconia and alumina was evaluated using zeta potential and sedimentation measurements. The microstructural, physical, and mechanical properties of the sintered components were analyzed in details using scanning electron microscopy (SEM), X-ray diffraction (XRD), density, shrinkage, and Vickers micro hardness measurements.

## 2. Experimental Section

### 2.1. Manufacturing Process

**Figure 1:**
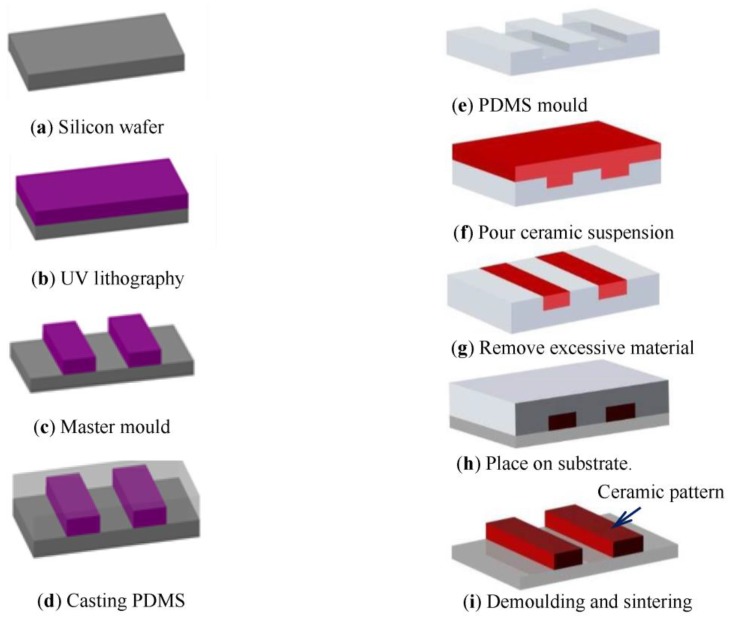
A schematic diagram of the manufacturing process.

The proposed process for manufacturing of YSZ/Al_2_O_3_ nanocomposite micro-parts is based on modified micro moulding soft lithography as shown in Figure 1. SU-8 is a commonly UV sensitive resin that used in nano/micro fabrication whereby the parts exposed to UV light become cured, while the rest of the layer remains soluble and can be washed away, resulting in high quality patterns. The SU-8 master mould is prepared using UV lithography. 4.9 mL of SU-8 2050 (Microchem, Westborough, MA, USA) was poured to a Silicon wafer to produce a 1 mm thick layer and that was baked on a hot at 65 °C. UV exposure was carried out using Canon PLA-501 mask aligner (Tokyo, Japan). The post baking was carried out on a hot plate again at 65 °C for 15 min and at 95 °C for 25 min. Afterwards, the wafer was washed with SU-8 developer in ultrasonic bath for 1 h. Next, the master mould was placed in an aluminium foil container. To prepare the soft mould, PDMS precursor was mixed with the curing agent (Dow Corning Corp., Midland, MI, USA) in a glass beaker with a weight ratio of 10:1 and degassed for 30 min. The PDMS mix was poured over the master mould and placed in a vacuum again until all residual bubbles were removed before it was cured at 65 °C for 4 h. When the soft moulds were cooled down to room temperature, the cured PDMS was gently peeled off from the SU-8 master moulds template. The desired micro-parts are micro-gears with a minimum tooth width of 75 µm, thickness of 1000 µm, and aspect ratio of 10. These dimensions were the smallest that the authors could achieve. The soft moulds are filled with the nanocomposite suspension and the excessive materials were removed using a stainless steel blade and placed on a PDMS substrate. Finally, mould left to dry and the green body are manually demoulded with the help of a micro tweezer and sintered to produce the micro-parts. Further details can be found in the literatures [21,22,23].

### 2.2. Suspension Preparation

Duramax D-3005 (ROHM and HAAS, Philadelphia, PA, USA) was mixed with distilled water and used as a dispersant. Powders of both YSZ with Yttria concentration of 5% (UCM, Berlin, Germany; average particle size of 0.4 µm) and Al_2_O_3_ (Sigma Aldrich, Gillingham, UK; average particle size of 0.32 µm) were added and mixed separately with the water mixture using mechanical stirrer for 180 min. Subsequently, Duramax B1007 and B1000 (ROHM and HAAS, Philadelphia, PA, USA), were mixed with the mixture. Finally, the suspension was vacuum degassed before getting poured into the PDMS mould to fill the mould cavities. Six nanocomposite samples were prepared in these experiments with Al_2_O_3_ contents of 0, 1, 2, 3, 4, and 5 vol.%. The theoretical density of the nanocomposites produced was determined using the rule of mixture.

### 2.3. Characterization

Sedimentation measurements and Zeta potential were studied to characterise the stability of the ceramic suspensions with regard to the pH and the dispersant concentration. Achieving a high zeta potential is important for the electrostatical stabilization of the aqueous suspensions, which is required to ensure a uniform dispersion of alumina powder into the YSZ matrix. It is also an essential step to obtain high green density components with homogenous composition and accurate dimensions. This could be secured by generating solid repellent forces between the powder to resist the Van der Walls forces and avoid accumulation. In the current experiments, zeta potential of the suspensions was measured using micro electrophoresis apparatus (Malvern Instruments, Malvern, UK). Prior to the measurement suspensions were ultrasonically dispersed for 8 min in order to improve particle dispersion and remove any bubbles. Zeta potential of each suspension was measured five times and the mean value was calculated. Sedimentation behaviours of both YSZ and alumina aqueous suspensions were also measured to obtain the optimum conditions of the dispersant content. The test is a direct method to observe the stability of ceramic solutions. Here, 4 mL of de-ionised water were stirred with the ceramic powders using a mechanical stirrer for 1 h. Then, the mixture was poured into a graduated cylinder of 25 mL capacity and the height ratio of the solution was calculated with regards to the pH values and dispersant concentration.

The apparent density of the sintered micro components was measured following Archimedes’ method by placing the sample into a beaker of ethanol. The micro hardness of the sintered micro components was carried out using the indentation technique (Buehler, Uzwil, Switzerland, model MicroMet 5100 Series Microindentation) with an indent load of 9.8 N according to ASTM C1327. Back-scattered electrons microscopy and energy dispersive X-ray spectroscopy (JEOL 7000, Peabody, MA, USA) analysis were carried out to characterise each of the Al_2_O_3_ and zirconia particles in the nanocomposite. Also, X-ray diffraction was applied to identify the different crystalline phases of YSZ/Al_2_O_3_ nanocomposite in order to understand the influence of the Al_2_O_3_ addition on the phase evolution of YSZ using Bruker D8 Autosampler-UK (Bruker, Germany). The test was carried out at 25 °C temperature by scanning steps of 0.014° (2θ) over a 24° < 2θ < 60° angular range and using Cu Kα radiation (λ = 1.5406 Å). In addition, EVA (Bruker-AXS) + 2009 (Powder Diffraction File, ICDD (International Centre for Diffraction Data)) were used as phase ID software.

## 3. Results and Discussion

**Figure 2:**
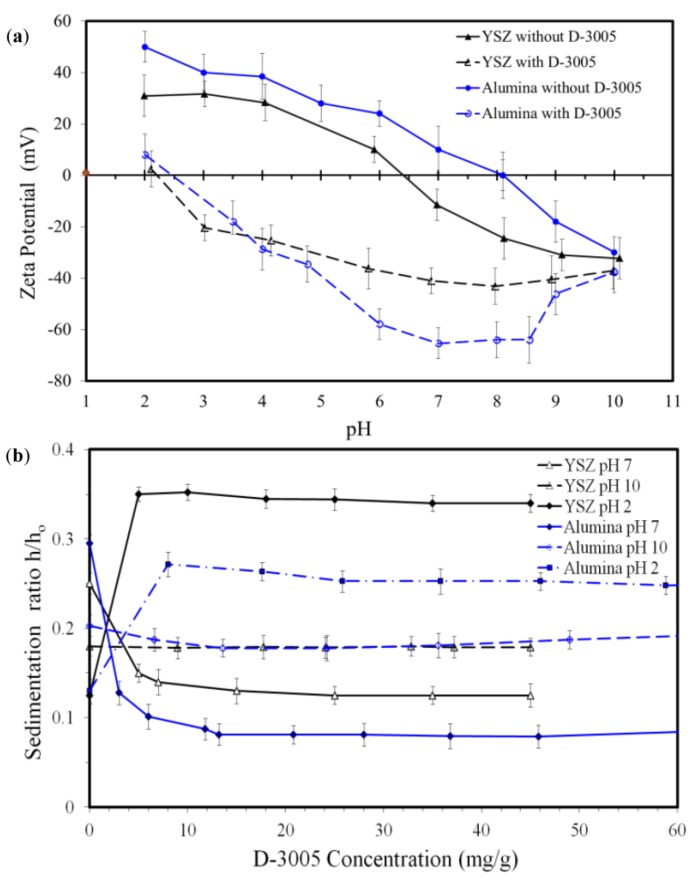
(**a**) Zeta potential and (**b**) sedimentation measurements with respect to pH and dispersant concentration for both YSZ and alumina.

Zeta potential of both YSZ and Al_2_O_3_ powders are presented in Figure 2a. For YSZ samples, the isoelectrostatic point (IEP) was found at pH_IEP_ = 6.4. Above and below this point, zeta potential was measured to be negative and positive respectively. When D-3005 was introduced, the magnitude of the surface charge on YSZ particles has been increased and remained negative over the studied range of pHs (from 2 to 10). In addition and as seen in Figure 2a, the pH_IEP_ was significantly decreased to reach 2.2. For alumina samples, zeta potential changed from +50 to −30 mV as the pH of the suspension increased from 2 to 10, with a pH_IEP_ of 8.1. Again, the addition of D-3005 resulted in a substantial rise of zeta potential with a peak of about −65 mV in the pH range from 7.0 to 8.5. The pH_IEP_ has also been reduced to a value of about 2.5. From the above results, suspensions with pH values in the range 7.0–8.0 and with the addition of dispersant are believed to be the most highly dispersed ones and therefore, they were selected for the preparation of zirconia/alumina nanocomposite suspensions.

The sedimentation behaviour of aqueous YSZ and alumina suspensions as functions of dispersant concentration and pH values is shown in Figure 2b. It was aimed to optimize the dispersant concentration and pH values that would provide maximum stability. Therefore three suspensions were prepared with pH values of 2, 7 and 10, to assess the performance of the dispersant in acidic, neutral and alkaline media respectively. As shown in Figure 2b, when no dispersant was added the sedimentation height of YSZ suspensions was minimum at pH values of 2 and 10, while it was maximum at pH = 7. These results clearly showed that at pH values of either 2 or 10, and in the absence of a dispersant, the best dispersion could be obtained. These results could be reasoned by comparing the results presented in Figure 2a,b. In a neutral suspensions (pH = 7), zeta potential approached the IEP which was an indication of a very low value. A relatively small zeta potential of aqueous YSZ system indicates a higher possibility of agglomeration. Therefore, all particles of that suspension had the tendency to settle fast which resulted in larger sedimentation heights, see Figure 2b. On the other hand, at pH = 2 and 10, YSZ suspensions exhibited the best dispersion, which was indicated by the associated highest zeta potentials. With the addition of D-3005, and in the neutral conditions, the suspensions stability has been significantly improved and reached a peak at a concentration of 25.1 mg/g. No further rise of the settled height was recorded with the additional increase of D-3005 concentration. In an alkaline environment (pH = 10), introducing of D-3005 powder had no significant effect on the sedimentation heights. Conversely, addition of the dispersant to a suspension with a pH of 2 caused a significant rise of the height, reaching a maximum at a concentration of 10.1 mg/g. Further increase of D-3005 concentration had only caused a minor enhancement of the stability behaviour of the suspension. For the alumina suspensions, comparable results were obtained. In neutral conditions, the addition of D-3005 had a remarkable influence on the stability which achieved a maximum value at a concentration of 20.8 mg/g. Again, the settled height remained unchanged even with higher concentrations of D-3005. In addition, at pH = 10, sedimentation heights showed a minor decrease by addition of the dispersant, while they had rapidly raised, at a pH value of 2, reaching a peak value at a concentration of 8.1 mg/g. Upon further increase of D-3005 concentration, the suspension stability had been slightly enhanced.

**Figure 3:**
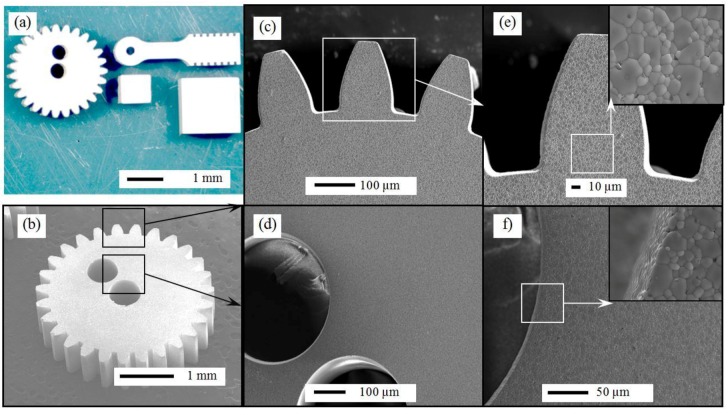
(**a**) Optical images of different green micro-parts. (**b**–**f**) scanning electron microscopy (SEM) images of sintered.

The optimum pH and dispersant concentration for both YSZ and alumina particle were used in the preparation of the green micro-parts, which were subsequently sintered at 1550 °C for 2 h. Figure 3 shows SEM images, at different magnifications, of the sintered micro-parts to allow a complete recognition of both the net shape quality and the dispersion of Al_2_O_3_ particles inside the zirconia matrix within micro-parts. Figure 3a shows the complete sintered micro gear. As shown, the micro gear retains all the micro features of the intended geometry eliminating the need of any further machining. Figure 3b,c show SEM images at higher magnification of the teeth and holes respectively, of the micro gear. The images clearly indicated the sharpness of gear edges and the homogeneity of the gear surface. At higher magnifications (about 400 times) the SEM images demonstrated homogenous nanocomposite structure without any visible flaws or agglomeration, as shown in Figure 3d,e. Moreover, no pores were detected within the micro-parts suggesting a high density of the fabricated micro part.

**Figure 4:**
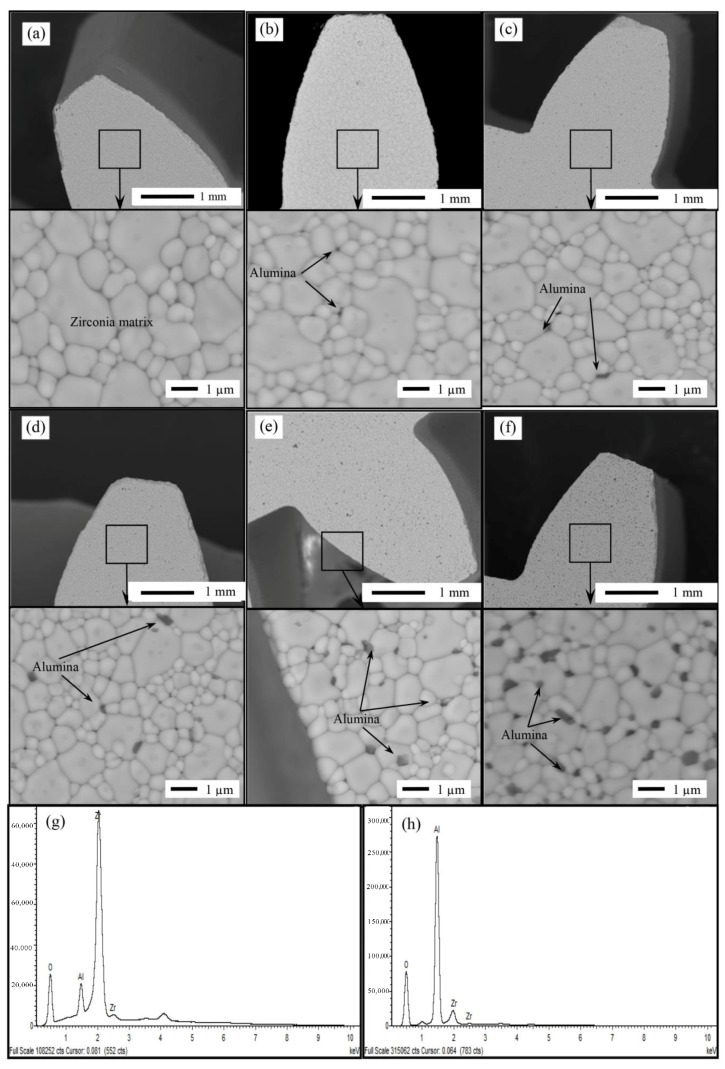
Back-scattered electrons (BSE) images of sintered YSZ/Al_2_O_3_ nanocomposite micro gear with different alumina nanoparticles content (**a**) 0 vol.%; (**b**) 0.2 vol.%; (**c**) 0.5 vol.%; (**d**) 1 vol.%; (**e**) 3 vol.%; (**f**) 5 vol.%; (**g**) EDS analysis of bright regions (zirconia); (**h**) EDS analysis of dark regions (alumina).

Back-scattered electrons (BSE) and energy dispersive X-ray spectroscopy (EDS) analysis were carried out to examine the surface of the nanocomposite and differentiate between alumina and YSZ particles in the structure. Figure 4 shows BSE images of YSZ/Al_2_O_3_ composites with different Al_2_O_3_ contents. The EDS analysis confirmed the identity of both the alumina (dark grains) and the YSZ (bright grains). It was found that with addition of 0.2% alumina, the BSE image started to show dark particles lightly dispersed within the composite (Figure 4b). Increasing the alumina concentration to 0.5% increased the darks spots in the BSE images (Figure 4c). As more alumina was added the density of black areas increased significantly within the YSZ matrix. See Figure 4d–f that showed BSE images of YSZ/Al_2_O_3_ composites with Al_2_O_3_ contents of 1%, 3% and 5% respectively. It was also noted that alumina particles were homogeneously distributed in YSZ matrix. In addition the diameter of alumina particles was determined to be of less than 1 µm in most of the cases, which reflects the success of the studied manufacturing technique in providing an excellent dispersion of the alumina particles and preventing their agglomeration. Furthermore, the addition of alumina did not affect the grain size or the dispersion quality, see Figure 4. Finally, it was observed that Al_2_O_3_ particles were mostly located at YSZ grain boundaries rather than inside the grains themselves.

**Figure 5:**
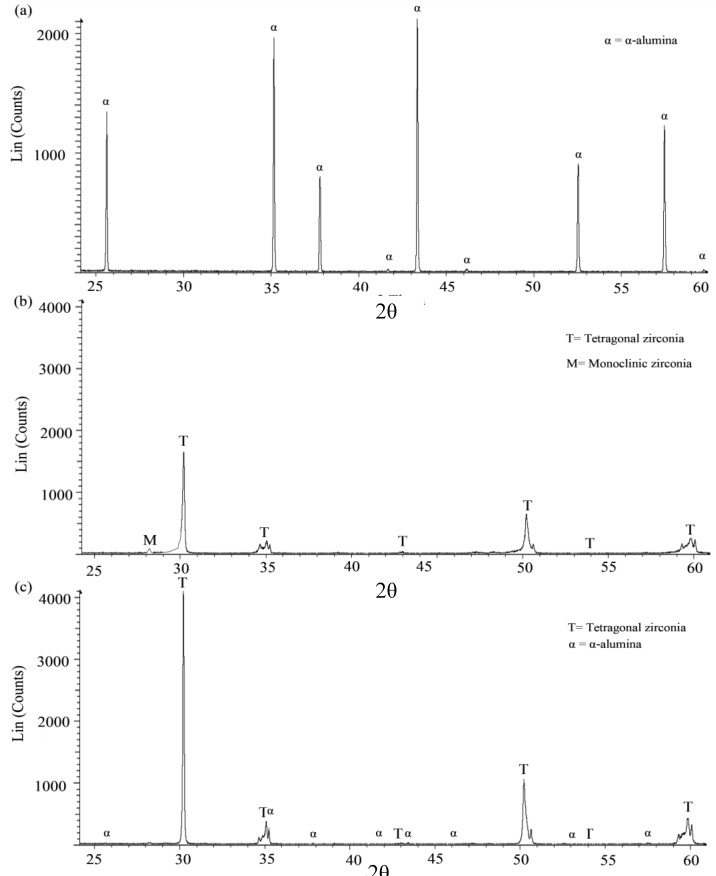
X-ray diffraction (XRD) patterns of the sintered (**a**) Alumina; (**b**) YSZ; and (**c**) 1 vol.% Al_2_O_3_.

The crystalline phases of YSZ/Al_2_O_3_ nanocomposite were examined via X-ray diffraction (XRD) to study the influence of Al_2_O_3_ addition on the YSZ phase transformation. Figure 5a shows an XRD image of a sintered alumina sample. The figure depicted that the only phase detected was the α alumina. Figure 5b shows X-ray diffraction results of a sintered YSZ sample, in which a tetragonal phase was identified as a dominating phase with traces of monoclinic phase as suggested by the observed low intensity signal at 2θ of 28°. The effect of adding 1 vol.% Al_2_O_3_ on the evolution of the nanocomposite phases is shown in Figure 5c which demonstrated a high intensity of the tetragonal phase with minor peaks of α alumina. No other phases were detected in the pattern. The peak intensities of the tetragonal phase in the nanocomposite were almost doubled with the addition of 1 vol.% alumina. This is clear when comparing the XRD patterns in Figure 5b,c. In addition, the analysis of YSZ/Al_2_O_3_ nanocomposite confirmed the existence of a pure tetragonal zirconia phase. Thus, it could be concluded that the introduction of Al_2_O_3_ to YSZ matrix can effectively eliminate the possibility of phase transformation of other zirconia phases such as the monoclinic phase.

**Figure 6:**
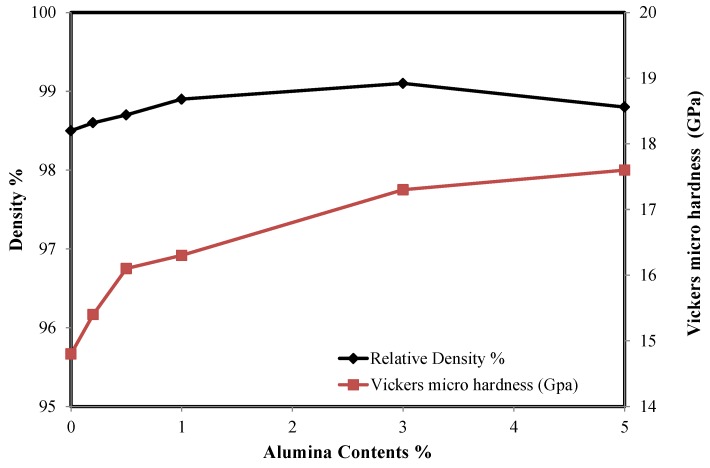
The sintered density and micro hardness of YSZ/Al_2_O_3_ composite as a function of alumina content.

Figure 6 shows plots of the sintered density and Vickers micro hardness of YSZ/Al_2_O_3_ nanocomposite micro components versus Al_2_O_3_ content. It can be observed that the addition of small amount of Al_2_O_3_ nano particles to the YSZ matrix had resulted in an obvious improvement of the sintered nanocomposite density, as shown in Figure 6. The relative sintered density of the YSZ micro part increased slightly from 98.5% to 99.1% because of the addition of 1 vol.% alumina. The enhanced density is suggested to be a direct effect of the increased green packing upon the addition of Al_2_O_3_ nano particles. However, a further increase of the alumina concentration (up to 5 vol.%) had caused a slight reduction in the density. It should be emphasized that the measured green density of monolithic YSZ powders and 1 vol.% of alumina YSZ nanocomposite were 55.9% and 56.7% respectively. In such particles mixture, the smaller particle size of alumina nano particles was able to occupy the vacancies between the large particles of the YSZ and hence increases the density.

In addition, Vickers micro hardness YSZ/Al_2_O_3_ nanocomposite was found to increase steadily with the addition of alumina nano particles. See Figure 6. The micro hardness of pure YSZ sintered at 1550 °C was about 14.65 GPa which was increased to 17.6 GPa when 5 vol.% Al_2_O_3_ were added to the zirconia matrix. Such improvement is believed to be due to the increased sintered density. Higher density means fewer pores and hence strengthens the material and improves hardness. The improvement was also because of the remarkable hardness properties of the alumina contents when compared to YSZ. Therefore, the greater the hardness of Al_2_O_3_ nanoparticles, the better the resistance to plastic deformation and the higher the wear resistance, which are key properties of micro gears whose surfaces are often subject to contact loads.

## 4. Conclusions

In this study, net shape YSZ/Al_2_O_3_ nanocomposite micro-components were successfully manufactured through a process based on soft lithography and powder dispersion. The results indicated that the addition of the optimum dispersant amounts and pH values when preparing zirconia and alumina suspensions were helpful in obtaining a well-dispersed nanocomposite. The results of X-ray diffraction of YSZ/Al_2_O_3_ nanocomposite micro components revealed the presence of pure tetragonal zirconia phase, and the ability of the addition of alumina to eliminate the presence of monoclinic phase. It was also shown that the addition of Al_2_O_3_ nano particles had significantly improved micro hardness of the nanocomposite. The sintered YSZ/Al_2_O_3_ micro components were shown to achieve a sintered density and micro hardness of 0.4% and 17% respectively, greater than the pure YSZ sintered micro components. The fabricated nanocomposite is therefore suggested to be an ideal material to replace pure ceramic materials formerly adopted for the fabrication of ceramic micro components.
